# Correlation among Antioxidant, Antimicrobial, Hemolytic, and Antiproliferative Properties of *Leiothrix spiralis* Leaves Extract

**DOI:** 10.3390/ijms13079260

**Published:** 2012-07-24

**Authors:** Marcelo Gonzaga de Freitas Araújo, Felipe Hilário, Wagner Vilegas, Lourdes Campaner dos Santos, Iguatemy Lourenço Brunetti, Claudia Elena Sotomayor, Tais Maria Bauab

**Affiliations:** 1 Biological Sciences Department, Faculty of Pharmaceutical Sciences, São Paulo State University—UNESP, Araraquara, SP 14801-902, Brazil; E-Mail: tmbauab@fcfar.unesp.br; 2 Organic Chemistry Department, Chemistry Institute, São Paulo State University—UNESP, c.p. 355, Araraquara, SP 14800-900, Brazil; E-Mails: felipehilario1e6@hotmail.com (F.H.); vilegasw@gmail.com (W.V.); loursant@iq.unesp.br (L.C.S.); 3 Clinical Analysis Department, Faculty of Pharmaceutical Sciences, São Paulo State University—UNESP, Araraquara, SP 14801-902, Brazil; E-Mail: brunetti@fcfar.unesp.br; 4 Department of Clinical Biochemistry, CIBICI-CONICET, Faculty of Chemical Sciences, National University of Cordoba, Haya de la Torre y Medina Allende, Córdoba 5000, Argentina; E-Mail: csotomay@fcq.unc.edu.ar

**Keywords:** *Leiothrix spiralis*, luteolin, antioxidant, antimicrobial, hemolytic, citotoxicity

## Abstract

The biological activities of a plant extract depend on a complex sum of individual properties including the antioxidant activity. Several biological activities protect against the harmful action of reactive oxygen species (ROS), and here we focused our attention on the relationship between the biological activities tested and the antioxidant properties. In this study, the total flavonoid content as well as the antioxidant, antimicrobial, hemolytic and cytotoxicity activities of the methanolic extract of *Leitothrix spiralis* leaves were evaluated. The extract showed a total flavonoid content of 19.26% and the chemical characterization by HPLC-PAD confirmed the presence of flavonoids as the major secondary metabolite compounds. Significant antioxidant activity (IC_50_ = 1.743 μg/mL ± 0.063) was demonstrated and was effective against Gram-negative organisms and all Candida strains tested, and showed an ability to inhibit hyphal formation. Non-hemolytic and antiproliferative activity could be demonstrated.

## 1. Introduction

Reactive oxygen species (ROS) and reactive nitrogen species (RNS), including free radicals such as superoxide radical anion, hydroxyl radicals, singlet oxygen, hydrogen peroxide and nitric oxide are continuously produced in human cells [1,2]. In this sense, natural products have been attracting scientific interest due to their antioxidant and chemopreventive properties. It is well-known that one of the main characteristics responsible for the antioxidant activity of a plant extract is its high content of phenolic compound and its ability to scavenge free radicals, which can play a part in the protection against the harmful action of ROS. Phenolic compounds also exhibit a wide range of other biological effects, such as anti-viral, antibacterial, and anti-tumor [3–7].

Oxidative stress is now recognized as a major factor associated with the development of chronic diseases, including cancer and cardiovascular disease. This has led to the hypothesis that the beneficial effects of natural products could be largely explained by their high content of antioxidants [8,9]. Alterations in the regulation of a number of key pathways in controlling cell proliferation are necessary for the establishment of all tumors. Several antioxidants in plants have been suggested to contribute to the anticarcinogenic effect, and others such as flavanols have also been able to inhibit cancer cell proliferation *in vitro* [10].

*Leiothrix*, a Eriocaulaceae genus, is exclusively found in South America of which 37 species are mainly restricted to Brazil. Some species of this family are of great economic importance since they are exported as ornamental plants to various countries, mainly Germany and Japan [11]. Chemical studies of the contents of soluble phenolic compounds of Leiothrix species were performed and the presence of xanthones and flavones, including nepetin-7-*O*-β-d-glucopyranoside, nepetin-7-*O*-β-d-arabinopyranoside and luteolin *O-* and *C-*glucopyranoside, rutin, 6-methoxyapigenin-7-*O*-β*-*d*-*glucopyranoside and 6-methoxyapigenin, were identified [5,12–14]. Antimicrobial activity of phenolic compounds from *Leiothrix spiralis* was investigated, showing activity on different strains of bacteria and yeasts [15]. Interest in natural antioxidant sources prompted us to investigate the biological activity of *L. spiralis*.

Although many studies concerning the antioxidant activity of plant extracts have been performed, only a few have tried to correlate this activity with antimicrobial and antiproliferative properties. Thus, the aim of the present study was to chemically characterize and quantify total flavonoids in the methanolic extract of *L. spiralis* leaves, as well as to evaluate its antioxidant, antimicrobial, hemolytic and human cancer cell antiproliferative activities.

## 2. Results and Discussion

### 2.1. Chemical Characterization

**Figure 1:**
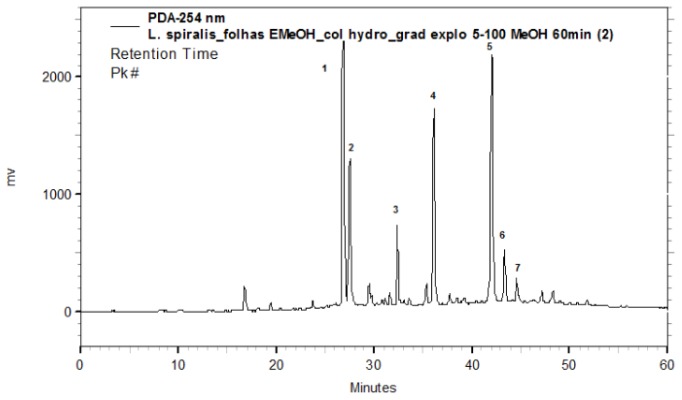
HPLC-PAD (Photodiodo Array Detector) chromatogram recorded at 254 nm of the methanolic extract of leaves from *L. spiralis*. For chromatographic conditions see Experimental section.

**Figure 2:**
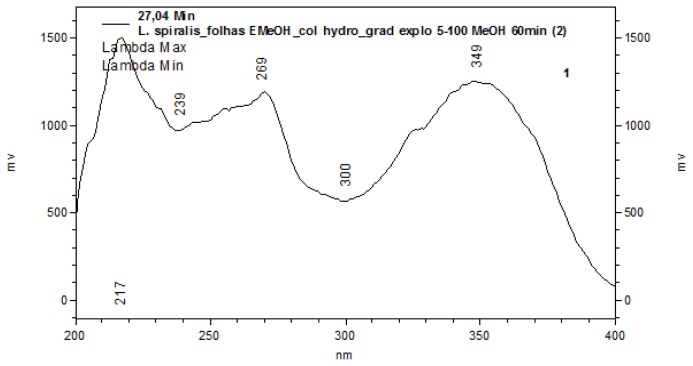

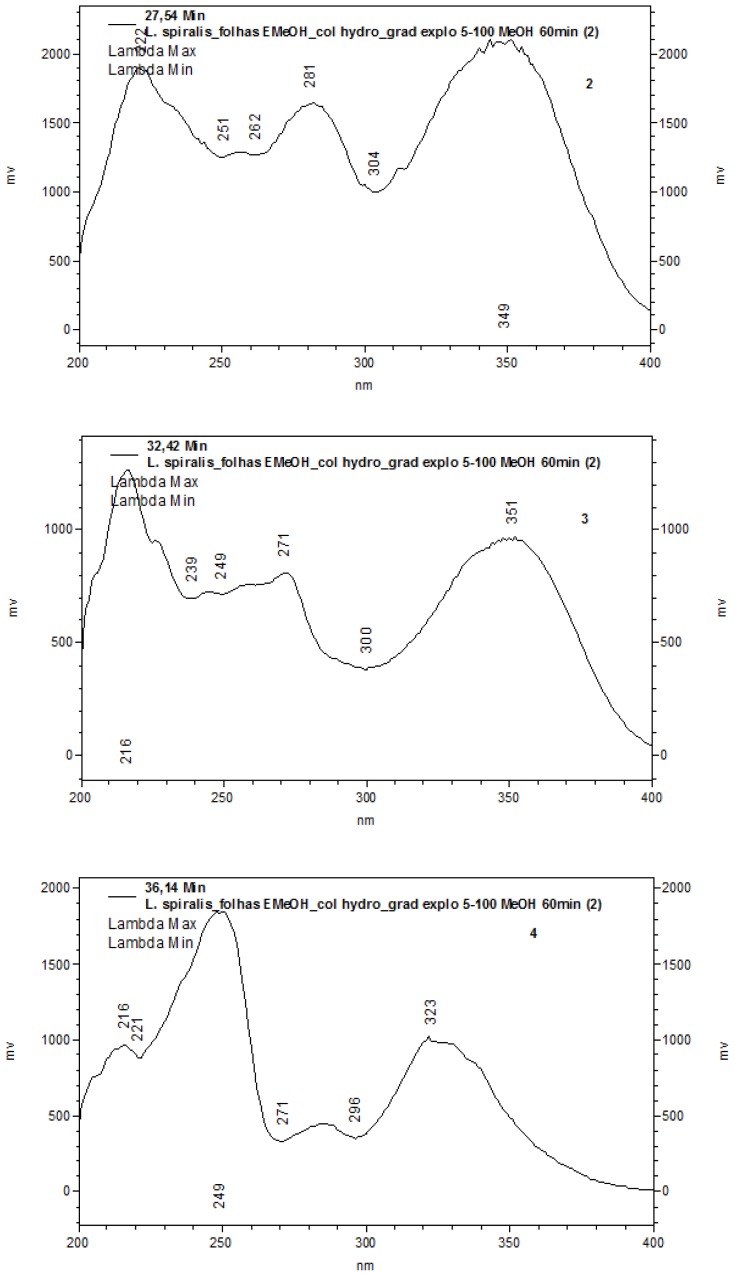

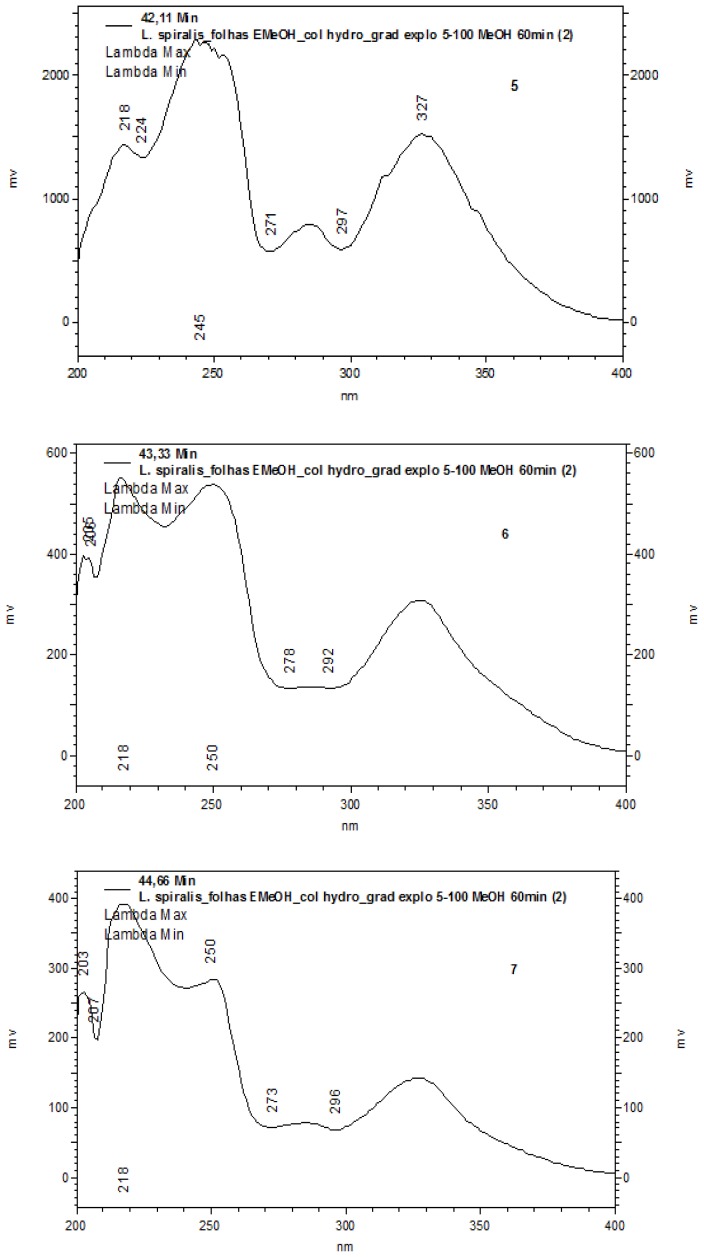
UV spectra of the chromatographic (HPLC) data of the methanolic extract of leaves from *L. spiralis* (peaks 1–7). Conditions: Phenomenex Synergi Hydro RP18 (250 × 4.6 mm i.d.; 4 μm) equipped with a Phenomenex security guard column (4.0 × 2.0 mm i.d.). The mobile phase composition was methanol. The gradient program was as follows: 5%–100% MeOH in 60 min. The flow rate was 1.0 mL/min and the total run time was 60 min.

The characterization of analytes was based on the comparison of the UV spectra of the peaks (Figure 1). With PAD (Photodiodo Array Detector) scanning from 200–600 nm, it was possible to obtain the UV spectra of each peak, which permitted the selection of a suitable wavelength in order to maximize the detection of the constituents. According to their corresponding UV absorbance, a wavelength of 254 nm was determined to be the most appropriate to ensure maximum detection for the simultaneous analyses of all compounds present. The UV spectra of the peaks corroborated the presence of flavone derivatives (peaks 1 to 7 with bands at 239–270 nm and 327–349 nm) [16] (Figure 2).

### 2.2. Total Flavonoids

The total flavonoid content was calculated based on the luteolin analytical curve and the percentage composition of flavonoid in the extract was 19.26 ± 0.04%. These results indicate the presence of significant amounts of that class of phenols.

### 2.3. ABTS Radical Cation Scavenging Activity

Table 1 shows the concentrations of the extract needed to decrease the initial ABTS (2,2′-azinobis-(3-ethylbenzothiazoline-6-sulfonic acid)) concentration by 50% (50% Inhibitory Concentration, IC_50_). The antioxidant activity of the extracts was expressed in quercetin and luteolin equivalents by comparing IC_50_ of the extracts with IC_50_ of standards. The IC_50_ value of the extract showed significant antioxidant activity when compared to standard quercetin and luteolin.

### 2.4. Antibacterial and Antifungal Susceptibility

The results of minimal inhibitory concentration (MIC) determinations of the antimicrobial activity (Table 2) showed noticeable MIC values for the methanolic extract of leaves from *L. spiralis*, against *Candida* species and Gram-positive bacteria. Luteolin showed activity against all microorganisms tested. The results of minimal fungicidal concentration (MFC) determination indicated that the fungicidal effect of the extract on the studied microorganisms could be expected. A close look at MFC and MIC values revealed that most MFC values correspond to MIC values.

### 2.5. Inhibition of Hyphal Formation

**Figure 3:**
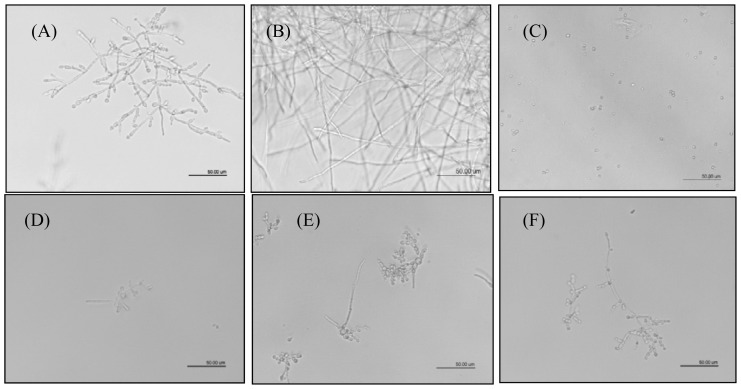

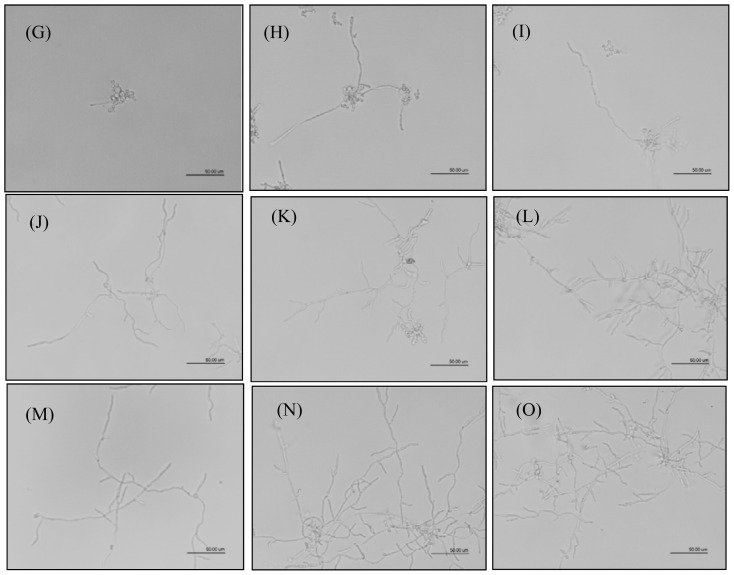
Hyphal formation of *C. albicans* NCPF 3153 cells. (**A**) 12 h normal growth; (**B**) 24 h normal growth; (**C**) *C. albicans* cells treated with 0.005 mg/mL anphotericin B (ANF) as the positive control; Hyphal formation of *C. albicans* cells was obviously inhibited by the extract at (**D**) 1 mg/mL; (**E**) 0.5 mg/mL; (**F**) 0.25 mg/mL at 12 h; and (**G**) 1 mg/mL; (**H**) 0.5 mg/mL; (**I**) 0.25 mg/mL at 24 h. Luteolin did not inhibit the hyphal formation (**J**) 0.25 mg/mL; (**K**) 0.125 mg/mL; (**L**) 0.062 mg/mL at 12 h; (**M**) 0.25 mg/mL; (**N**) 0.125 mg/mL; (**O**) 0.062 mg/mL at 24 h, compared with (**A**) and (**B**). The black bar represents a length of 50 μm.

*C. albicans* cells were incubated for 12 and 24 h in the presence of three concentrations of extract based on MIC values to *C. albicans*, and anphotericin B (ANF) at 5 mg/L as positive control, and then observed under an inverted light microscope (Figure 3). In the absence of the drugs, hyphal formation was observed, while in the presence of the highest concentration of extract tested, hyphal formation of *C. albicans* was significantly inhibited. The different concentrations of luteolin were not able to inhibit hyphal formation.

### 2.6. MTT and LDH Cell Viability

**Figure 4:**
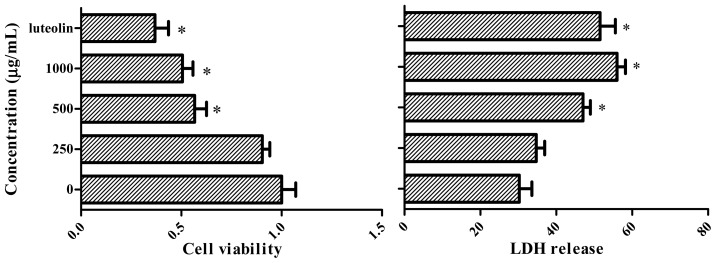
Effect of extract on cell viability and LDH release in cultured HeLa cells. Cells were treated with different concentrations of extract, and luteolin (31 mg/L) was used as positive control.* Significantly different from the basal conditions.

Figure 4 shows the effect of the extract on the cell viability of human cervical adenocarcinoma cells line (HeLa cells) after a 24 h incubation period. Luteolin significantly inhibited cell growth at the tested concentration (*p* < 0.05), and cell viability was affected by the extract treatment at 500 and 1000 mg/mL when compared with untreated cells. Cell injury was quantitatively assessed by the measurement of lactate dehydrogenase (LDH) release. After a 24 h incubation period, luteolin and the treatment with different extract concentrations significantly increased cell LDH release (*p* < 0.05), when compared with the untreated control.

### 2.7. Hemolytic Assay

**Figure 5:**
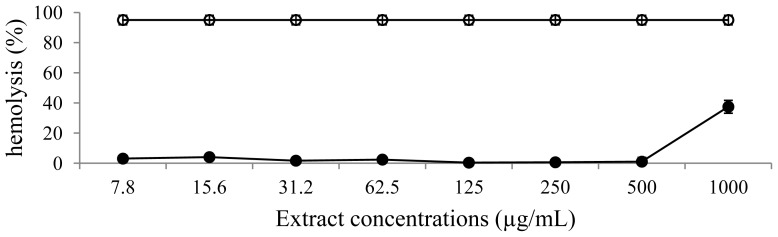
Hemolytic activity of extract of leaves of *L. spiralis.* Extract (filled circle) and positive control Triton X-100 (open circle).

**Figure 6:**
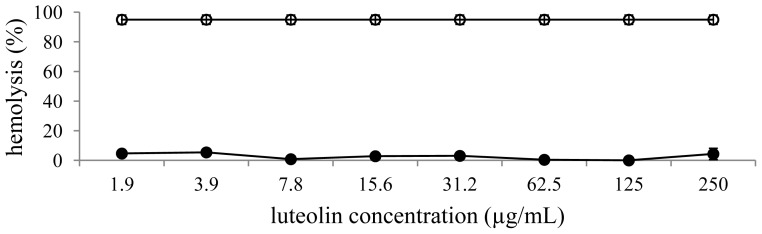
Hemolytic activity of luteolin. Luteolin (filled circle) and positive control Triton X-100 (open circle).

Figures 5,6 show the hemolytic activity of the extract investigated by measuring the lysis of a 10% (*v*/*v*) human red blood cells suspension in a spectrophotometric assay. In this experiment, Triton X-100 1% (*v*/*v*) was used as a positive control and induced 95.0 ± 3.1% of red blood cell lysis. The extract and luteolin showed no significant effect on red blood cells lysis.

ROS and free radicals such as superoxide anion, hydrogen peroxide and hydroxyl radicals are considered to be implicated in degenerative processes related to aging, cancer and atherosclerosis, mainly because they can induce the oxidative damage of cell membranes, DNA, and proteins [17]. Thus, blocking the generation of ROS and free radicals by supplementation of antioxidants might have a beneficial role in preventing these free radical-related diseases. Several studies have shown that phenolics are the bioactive compounds that offer more benefits to human health and many authors have reported a direct relationship between total phenolic content and antioxidant activity in numerous seeds, fruits and vegetables [18–20]. As expected, methanolic extract obtained from leaves of *L. spiralis* presented high flavonoid content. This fact correlates with the remarkable differences in the polarity of the extraction solvents used and the solubility of phenolic compounds in them. In fact, methanol (polar solvent) is considered one of the best solvents for phenolic extraction. The chemical analysis of the extract by HPLC-PAD confirmed the presence of flavonoids as the major secondary metabolite compounds in leaves of *L. spiralis*. The features of the UV spectra with bands of maximum of absorption at 239–250 (Band II) nm and 327–349 (Band I) nm suggested the presence of flavones [16]. Some authors studied the contents of soluble phenolic compounds of the capitula of 21 species of *Leiothrix*. The presence of luteolin *O*- and *C*-glucopyranoside was determined in *Leiothrix* species [13] and another study verified the presence of xanthones and flavone with antioxidant activity in capitula of *L. flavescens* and *L. curvifolia* [14]. In agreement, in this study the IC_50_ values of methanolic extract obtained from the ABTS assay were significantly correlated with the total flavonoid standards, suggesting that flavonoids in these extracts constitute an important portion of the antioxidant activity.

It was suggested that antioxidant capacity of *L. spiralis* was mainly due to the presence of luteolin derivatives. Multiple mechanisms may underlie the antioxidant effect of luteolin. Luteolin functions as a ROS scavenger through its own oxidation and possesses the essential structures to antioxidant activity of flavonoids: 3′,4′-hydroxylation, the presence of a double bond between carbons 2 and 3 and a carbonyl group on carbon 4. The hydrogen atom from an aromatic hydroxyl group can be donated to free radicals. As an aromatic compound, luteolin can support unpaired electrons around the electron system. Moreover, luteolin inhibits ROS generating oxidases and may directly inhibit enzymes that catalyze the oxidation of cellular components [21].

Flavonoids exhibit inhibitory effects against multiple microorganisms. The antimicrobial actions result from an interaction between these compounds and the cell membrane of the target microorganisms, probably due to their ability to bind with extracellular and soluble proteins and also with cell walls [22]. In this study, the extract showed antimicrobial activities against Gram-positive organisms. These differences may be attributed to the fact that the cell wall in Gram-positive bacteria is of a single layer, whereas the Gram negative cell wall is a multilayered structure. This tendency could be explained by the fact that Gram negative bacteria possess an outer membrane surrounding the cell wall, which restricts diffusion of bioactive compounds due to the presence of lipopolysaccharide [23]. However, phenolic compounds maybe not only affect bacterial membranes but also mammalian cells such as erythrocytes [24]. They can interact with membrane lipids and proteins of erythrocytes and result in membrane damage [25]. The *in vitro* toxicity on the red blood cell membrane of various plant extracts has been studied and correlated with their constituents. The extract and luteolin showed very low hemolytic activity against human blood cells. On the other hand, flavonoids have been revealed to protect biological membranes against free radical-induced oxidative damage. They scavenge reactive oxygen species and, in some cases, their interaction with cellular proteins has been suggested, especially with heme proteins, which exert their physiological functions by the oxidation and reduction of heme iron [26–28].

In a previous work, chemical fractionation of the methanolic extract of leaves of *L. spiralis* Ruhland afforded the flavonoids luteolin-derivatives and xanthones with antimicrobial activity [15]. The methanolic extract of leaves from *L. spiralis* as well as the flavonoid luteolin, showed activity against all Candida species tested. *C. albicans* is a dimorphic yeast and its ability to switch from yeast cells to hyphae is considered to be important for the interactions of *C. albicans* with its host. Hyphae are an important factor of fungal virulence. It is through hyphae that *C. albicans* invades human tissues [29]. The microscopy observations showed that extract at a concentration of 1000 μg/mL clearly inhibited hyphal formation during the hyphal induction of *C. albicans*. This result is important to characterize the action of the extract on the yeast tested. The antifungal activity of the phenolic compounds has been attributed to their lipophilic properties, which determine their ability to penetrate into the plasma membrane and induce changes in the physicochemical properties of the cell wall, cell membrane, and cellular organelles [30]. Antioxidant activity may be important in the antifungal mode of action of phenolic compounds. A hypothesis is that during stress, fungi respond by controlling secondary metabolite production and authors have also linked the antioxidant activity of phenolic compounds to their activity on metabolites biosynthesis [31–32].

The human body possesses multiple endogenous systems to protect cellular molecules against the oxygen radical-induced damage. These defense mechanisms include antioxidative enzymes, such as superoxide dismutase (SOD); catalase; and glutathione peroxidase [33]. Mammalian cell culture and cell-free studies have begun to elucidate the underlying mechanisms of the potential protective effects of plant constituents against carcinogenesis, including antimutagenic effects of extracts and natural compounds against cancer inducing agents. Cells are very susceptible to mild oxidation, for example, with dilute solutions of hydrogen peroxide. These changes can be protected by additions of various antioxidant compounds such as flavonoids. Therefore, it is possible to speculate that flavonoids should offer cancer protection via their antioxidant properties [34].

Antioxidant activity is involved in cancer prevention at the initiation stage while antiproliferative activity is targeting cancer cells at the promotion and progression stages. However, it should be noted that by using the MTT-assay, it is not possible to differentiate between cell growth inhibition and an increase in cell death [35]. In the meantime, the LDH leakage assay revealed toxicity after exposure at the same concentrations, therefore, the extract has promoted rupture of the cell membrane in HeLa cells. In this regard, data from studies focusing on the elucidation of the molecular basis of the putative anticancer activity of polyphenols indicate that both, the inhibition of cell growth and the induction of cell death, play a role in the antitumor activity of polyphenols [35–37]. Furthermore, data from other cell culture studies strongly suggest that the mechanism whereby phenolic compounds modulate cell proliferation is remarkably dose-dependent [21–38]. Interestingly, the arrest of HeLa cell growth was evident after a 24 h treatment, with the highest concentration used. This indicates that the polyphenols in the extract promptly initiated a series of cellular events leading to the inhibition of cell proliferation and/or the induction of cell death.

## 3. Experimental Section

### 3.1. Plant Material

Leaves of *L. spiralis* (Bong.) Ruhl. were collected in May 2006 in Diamantina, Minas Gerais State, Brazil, and authenticated by Dr. Paulo Takeo Sano from the Institute of Biosciences of the University of São Paulo (IB-USP), São Paulo. A voucher specimen (SANO n° 4798) was deposited at the Herbarium of the IB-USP. Leaves of *L. spiralis* were oven dried at 45 °C for one week and powdered separately. The dried leaves of *L. spiralis* (2.00 g) were powdered and extracted with hexane, methylene chloride, and methanol, successively. The methanol extract (1.2 g) was used for this work. Luteolin was obtained by Sigma^®^.

### 3.2. Chemical Characterization of the Extract

The chromatographic profile of the methanolic extract of the leaves from *L. spiralis* was obtained by using a Jasco (Tokyo, Japan) liquid chromatograph equipped with a PU-2089 Plus pump, a MD-2010 Plus Photodiodo Array detector (PAD) and a Rheodyne 7725 sample injector with a 20 μL sample loop. The analytical column was a Phenomenex Synergi Hydro RP18 (250 × 4.6 mm i.d.; 4 μm) equipped with a Phenomenex security guard column (4.0 × 2.0 mm i.d.). The mobile phase composition was methanol. The gradient program was as follows: 5%–100% methanol in 60 min. The flow rate was 1.0 mL/min and the total run time was 60 min. The software EZChrom Elite 3.17 was used for control analytical system, data collection and processing.

### 3.3. Total Flavonoids

Total flavonoids were estimated according to Georgetti S.R. *et al*. [39]. To 0.5 mL of sample, 0.5 mL of 2% AlCl_3_ ethanol solution was added. After 60 min at room temperature, the absorbance was measured at 351 nm. Total flavonoid contents were calculated as luteolin equivalent from an analytical curve.

### 3.4. ABTS Radical Cation Scavenging Activity

The free radical scavenging activity of the extract was determined by ABTS radical cation decolorization assay [40]. It involved the generation of ABTS^•+^ chromophore by the oxidation of ABTS with potassium persulfate. The ABTS^•+^ radical cation was generated by reacting ABTS and potassium persulfate after incubation at room temperature in the dark for 12–16 h. The solution was then diluted by mixing 1 mL ABTS^•+^ solution with methanol, to obtain an absorbance of about 0.7 at 734 nm using the spectrophotometer. The reactive mixture (extract in different concentrations and ABTS^•+^) was allowed to stand at room temperature for 15 min and the absorbance was immediately recorded at 734 nm. Quercetin and luteolin standard solutions were prepared and analyzed under the same conditions. The results were expressed as 50% inhibitory concentration or IC_50_.

### 3.5. Antimicrobial Activity

The MIC was determined by the broth microdilution method, according to the standard reference method [41,42]. The antifungal activity was evaluated against *C. albicans* (ATCC 18804 and NCPF 3153), *C. krusei* (ATCC 6258), *C. parapsilosis* (ATCC 22019) and *C. tropicalis* (ATCC 750) and the antibacterial activity was evaluated against *Staphylococcus aureus* (ATCC 25923), *Bacilus subtillis* (ATCC 19659), *Enterococcus faecalis* (ATCC 29212), *Escherichia coli* (ATCC 25922), *Pseudomonas aeruginosa* (ATCC 27853) and *Salmonella setubal* (ATCC 19196). The extract and luteolin were dissolved in 25% methanol and water to initial concentration of extract of 1000 μg/mL. Then, a two-fold serial dilution was made in order to obtain concentration ranges of 7.8–1000 μg/mL. 100 μL of each concentration were added to 96-well microplates containing 80 μL of RPMI 1640 for yeast and Muller-Hinton broth for bacteria. The inocula of bacteria and yeast were standardized at 1.0 × 10^7^ and 2.5 × 10^3^ CFU/mL, respectively. Ciprofloxacin, fluconazole and 25% methanol and water were used as positive and negative control. The plates were incubated at 37 °C for 24 h for bacteria and 48 h for yeast. The assay was repeated three times. The MIC of the samples was detected after the addition (50 μL) of resazurin solution (0.2 mg/mL) for bacteria and 2.0% triphenyl-tetrazolium chloride (TTC) solution for yeast, and incubated at 37 °C for 30 min. Growth of bacteria changes the blue dye resazurin to a pink color. The pink color indicates positive growth, and blue indicates growth inhibition. Yeast growth changes the colorless TTC to a red color. MIC was defined as the lowest sample concentration that prevented this change and exhibited inhibition of microorganism growth.

For the determination of minimal bactericidal concentration (MBC) and MFC, a portion from each well that showed antibacterial activity and antifungal activity was plated on Muller-Hinton and Sabouroud agar, and incubated at 37 °C for 24 h. The lowest concentration that yielded no growth after this sub-culturing was taken as the MBC/MFC [43].

### 3.6. Inhibition of Hyphal Growth

*C. albicans* (NCPF 3153) cells from a 48 h stationary phase culture were transferred to microplate with RPMI 1640 medium supplemented with fetal bovine serum (FBS) to a final concentration of 2.5 × 10^3^ CFU/mL, and extract and luteolin solution were added to the growth medium to final concentrations of 0.5× MIC, 1× MIC and 2× MIC, and the cultures were incubated for 12 and 24 h at 37 °C, 5% CO_2_ [44]. The hyphal formation was observed under an inverted light microscope (Nikon TE 2000-U Eclipse) with the magnification of 400×. ANF (5 mg/L) was used as a positive control.

### 3.7. MTT and LDH Cell Viability Assay

Cell viability was determined by using the conversion of MTT to formazan via mitochondrial oxidation [45]. The human cervical adenocarcinoma cells line (HeLa—ATCC: CCL-2; 5000 cells/well) were treated with the extract and luteolin (0, 0.5× MIC, 1× MIC and 2× MIC to *C. albicans*) for 24 h. Then, MTT solution was added to each well at a concentration of 10 mg/mL and the plates were incubated at 37 °C for another 4 h. After incubation, 0.2 mL DMSO was added to each well to dissolve the formazan and the absorbance was read at 570 nm using a spectrophotometric microplate reader. The viability was determined based on a comparison with untreated cells. Luteolin was used as a positive (cytotoxic) control. The experiments were performed in triplicate and repeated at least three times.

Cell injury was quantitatively assessed by the measurement of LDH, released from damaged or destroyed cells, in the extracellular fluid 24 h after the experiment. An aliquot of bathing media was combined with NADH and pyruvate solutions. LDH activity is proportional to the rate of pyruvate loss. The quantity of LDH released by the cells into the medium was measured by the decrease in the absorbance at 340 nm for NADH disappearance within different times (0, 1, 2 and 3 min). The difference in absorbance per minute was determined and the average multiplied by the factor (10080).

The results were statistically analyzed by comparing the LDH and MTT values obtained at baseline with those obtained in different treatments by analysis of variance (ANOVA) followed by Dunnett test (multiple comparisons with one control) with *p* < 0.05.

### 3.8. Hemolytic Assay

Human erythrocytes from healthy individuals were collected in vacuum tubes containing heparin as anti-coagulant. The erythrocytes were harvested by centrifugation for 10 min at 2000 rpm and washed three times in phosphate buffered saline (PBS). To the pellet, PBS was added to yield a 10% (*v*/*v*) erythrocytes/PBS suspension. The 10% suspension was then diluted 1:10 in PBS. 0.1 mL of this suspension was added in triplicate to 96-well microplates containing 0.1 mL of extract (7.8–1000 μg/mL) or luteolin (1.9–250 μg/mL) serially diluted in PBS. Total hemolysis was achieved with 1% Triton X-100. The microplate was incubated for 1 h at 37 °C and then centrifuged for 10 min at 2000 rpm. The absorbance of the supernatant was measured spectrophotometrically at 450 nm [46]. The percentage of hemolysis was calculated and statistically analyzed by analysis of variance (ANOVA) followed by Dunnett test (multiple comparisons with one control) with *p* < 0.05.

## 4. Conclusions

The biological activity of a plant extract depends on a complex sum of individual properties including its composition, the existing compound structures, affinity for the target site, survival within the biological system, transport properties, and state of the target organism. In this study, we focused our attention on the relationship between some biological activities and antioxidant properties. The methanolic extract of *L. spiralis* leaves showed a significant antioxidant activity, and an antibacterial, antifungal, non-hemolytic and antiproliferative activity could be demonstrated. A better understanding of how antioxidant plant extracts inhibit microorganism growth and inhibit tumor cell proliferation will allow them to be more efficiently used and perhaps synergistically used with other antimicrobial, antiproliferative agents or their anti-hemolytic activity.

## Figures and Tables

**Table 1:** 50% Inhibitory Concentration (IC_50_) values of extract and standards obtained in the ABTS (2,2′-azinobis-(3-ethylbenzothiazoline-6-sulfonic acid)) radical test.

Samples	IC_50_ a

	Means ± SD
*L. spiralis* leaves	1.743 ± 0.063 b
quercetin	1.140 ± 0.038 b
luteolin	1.215 ± 0.031 b

a Values in μg/mL;

b <0.001; SD: standard deviation.

**Table 2:** Antibacterial and antifungal activity of the methanolic extract of *L. spiralis* leaves and luteolin. Minimal inhibitory concentration **(**MIC); minimal bactericidal concentration (MBC); minimal fungicidal concentration (MFC).

Microorganism	*L. spiralis*		Luteolin	

	MIC a	MBC/MFC a	MIC a	MBC/MFC a
*C. albicans*	500	1000	125	500
*C. krusei*	500	1000	250	250
*C. parapsilosis*	250	250	125	125
*C. tropicalis*	1000	1000	250	250
*S. aureus*	1000	1000	125	125
*B. subtilis*	1000	1000	62.5	125
*E. faecalis*	500	-	31.25	125
*E. coli*	-	-	250	250
*P. aeruginosa*	-	-	62.5	250
*S. setubal*	-	-	250	-

a Values of minimal inhibitory concentration given as μg/mL; (-) >1000 μg/mL.
